# Chronic Lipoid Pneumonia in a 9-Year-Old Child Revealed by Recurrent Chest Pain

**DOI:** 10.1155/2015/402926

**Published:** 2015-05-21

**Authors:** A. Hochart, C. Thumerelle, L. Petyt, C. Mordacq, A. Deschildre

**Affiliations:** ^1^Département de Pneumologie Pédiatrique, Hôpital Jeanne de Flandre, CHRU Lille, 59 037 Lille, France; ^2^Département d'Imagerie Médicale, Hôpital Calmette, CHRU Lille, 59 037 Lille, France

## Abstract

Lipoid pneumonia in children is a rare disorder due to accumulation of fatty oily material in the alveoli and usually associated with an underlying condition. In absence of obvious context, diagnosis remains difficult with nonspecific clinical and radiological features. We report the first case of voluntary chronic aspiration of olive oil responsible for exogenous lipoid pneumonia, in a previously healthy 9-year-old boy. Clinical presentation was atypical; LP was revealed by isolated chest pain. We discuss radiological and bronchial alveolar lavage characteristics suggestive of lipoid pneumonia. *Conclusion*. Lipoid pneumonia is a disease to be reminded of in children, which can occur with original findings in terms of etiology and clinical presentation.

## 1. Introduction

Lipoid pneumonia (LP) is a rare inflammatory disease of the lung due to accumulation of fatty oily material in the alveoli. The first description was nearly a century ago [1] but LP remains difficult to diagnose in the absence of obvious context.

Pediatric cases of exogenous LP happen especially with certain risk factors such as aspiration of large amounts of oily materials [2], mineral oil being the most frequent. This aspiration of fatty material induces a pulmonary inflammatory reaction with nonspecific clinical and radiologic features, similar to bacterial pneumonia, complicating or delaying the diagnosis.

We report a case of LP revealed by isolated chest pain, due to a voluntary chronic aspiration of olive oil in a child.

## 2. Observation

A 9-year-old boy was admitted in our pediatric department with recurring episodes of right chest pain. For one month, he had been complaining of intermittent spontaneous right chest pain happening once or twice a week. There was no other associated sign (no respiratory distress, fever, cough, or hemoptysis) and no history of thoracic trauma.

The child had no respiratory medical history and did not receive any medication. He was followed in a psychiatric unit for behavioral disorders with continuation of a normal schooling. Environment was healthy.

**Figure 1:**
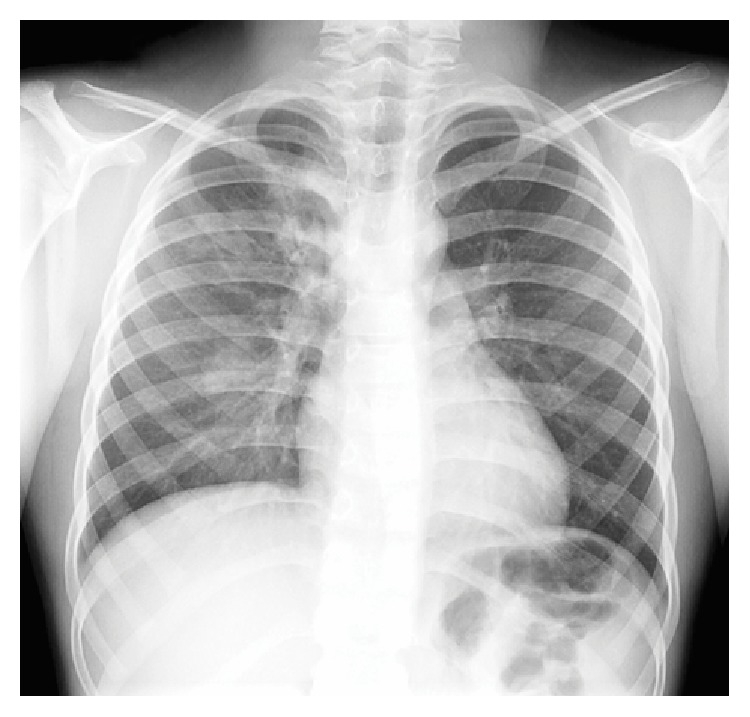
Initial chest X-ray: right upper and lower lobe infiltrations.

**Figure 2:**
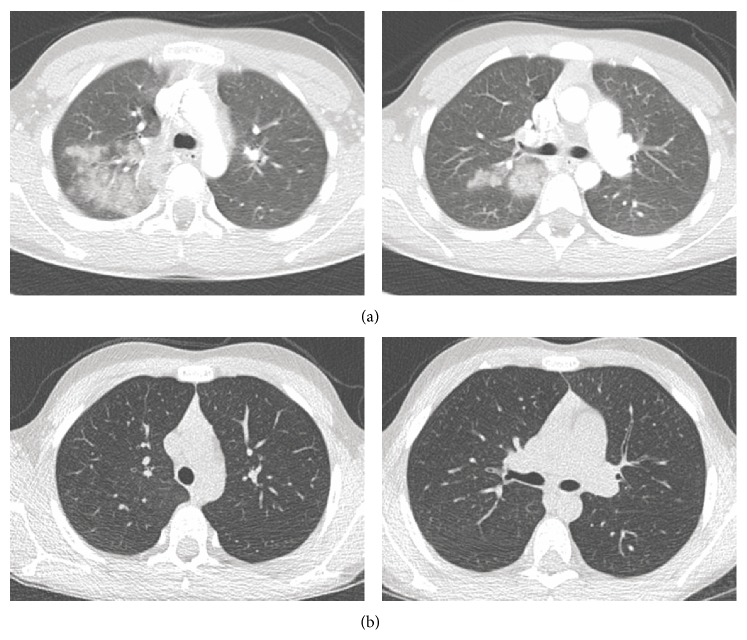
(a) Initial chest CT with upper and lower right lobe consolidations, characterized by low density (−129 HU within the lower lobe consolidation). (b) Chest CT two months later showed complete recovery.

On examination, his vitals were normal (oxygen saturation at 100% on room air, respiratory rate at 20/min). Chest auscultation and other clinical examinations were normal. Blood cells count, C-reactive protein, liver, renal, N-Terminal-Pro-B-Type Natriuretic Peptide, and troponin values were within normal limits. Cardiac evaluation was normal (electrocardiogram and transthoracic echocardiography). Chest X-ray revealed right upper and lower infiltrations (Figure 1). Chest CT showed airspace consolidations in the posterior segment of the right upper lobe and apical segment of the right lower lobe. These consolidations were characterized by very low density similar to fat tissue (Figure 2(a)).

Serological tests were positive for* Chlamydia pneumoniae* (IgM and IgG) and negative for* Mycoplasma pneumoniae*, toxocariasis, and ascariasis. Tuberculin skin test was negative. Based on the result of chlamydial serology, clarithromycin was introduced.

However the diagnosis of chlamydial infection was questioned because of the unusual clinical presentation (chest pains without fever and cough), the absence of biological inflammation, and the CT scan aspect of the consolidation (very low density). For these reasons, a bronchoscopy was performed during hospitalization and showed no abnormality. The bronchoalveolar lavage (BAL) was negative for all microorganisms (bacterial culture, viral real-time PCR) but showed evidence of inflammation with a high total cell count (2900 × 10^6^ cells/L) and increased lymphocytes (35%) and neutrophils (13%). Thirty percent of macrophages stained positive for lipid (lipid-laden alveolar macrophage (LLAM)). Exogenous LP was evoked.

The child improved during hospitalization and was discharged at day 3 without any residual pain. During the next week follow-up, the child and his family were asked about potential lipid aspiration, and we discovered the boy usually drank large amounts of olive oil when frustrated due to his behavioral disorders. Total eviction of oil was performed. One month later, the child had normal physical examination and did not report any pain. Chest CT showed complete recovery at 2 months (Figure 2(b)). The diagnosis of exogenous LP related to voluntary olive oil aspiration was retained.

## 3. Discussion

LP is an uncommon condition, due to accumulated lipids in the alveoli triggering a local inflammatory reaction. Exogenous forms are the most common, due to voluntary or accidental aspiration or inhalation of mineral, vegetable, or animal oil into the peripheral lung. Mineral oil, the most common incriminated substance, cannot be metabolized by pulmonary enzymes but is phagocytized by alveolar macrophages transforming in LLAM. The presence of LLAM triggers a granulomatous reaction in the alveoli, and chronic inflammation can lead to progressive pulmonary fibrosis [2].

In children, the main triggers of LP are accidental aspiration of mineral or vegetable oils, especially iatrogenic aspiration (laxative substances, oily nasal drops) [3, 4], and aspiration related to high-fat diet such as ketogenic diet [5]. Therefore, exogenous LP is usually associated with an underlying condition, such as severe gastroesophageal reflux, swallowing disorders, anatomical abnormalities of the pharynx and esophagus, cerebral palsy, or neuromuscular disorders [6]. These aspirations rarely occur in healthy patients. Psychological disorders responsible for LP are not described in children.

Excluding acute accidental aspiration of massive amount of lipids, diagnosis is difficult for chronic forms. Clinical symptoms are nonspecific; they vary from asymptomatic to severe presentation, depending on the duration of exposure, the type, and amount of aspirated fat. A previous study of exogenous LP in 28 children reported as main features: tachypnea (96%), cough (86%), and fever (82%) [2]. Other symptoms were dyspnea, lack of weight gain, and recurrent respiratory infections. On examination, main characteristics were crackles and wheezing, but 46% of children had a normal auscultation [2]. In adults, hemoptysis and chest pain have been also reported [6, 7]. Isolated chest pain as in our case is an unusual presentation of LP in children.

Without any obvious context, radiological characteristics and BAL are helpful to the diagnosis. High-resolution CT abnormalities of LP are consolidations, ground glass opacities, air-space nodules, and crazy-paving pattern [3, 8]. Lesions involve preferentially the upper right lobe with central and posterior distribution. The most typical CT finding is the unusual low density (−30 to −150 HU) within the consolidation area, suggesting the presence of fat [8]. The BAL is the reference method for diagnosis with presence of high count of LLAM. BAL fluid may have a macroscopic milky aspect with halo of supernatant fat. Cytological examination of our patient's BAL showed LLAM and marked inflammation with lymphocytes and neutrophils in relation with the local granulomatous reaction.

Except for the discontinuation of exposure, no other treatments are consensual in LP. In a prospective study of 10 children, Sias et al. demonstrated the potential role of multiple therapeutic BALs which facilitated the removal of LLAM implicated in the development of pulmonary fibrosis in chronic form [9]. Corticosteroids have also been tried in severe presentation and may be effective on the inflammatory response and would possibly prevent pulmonary fibrosis [9].

Prognosis of exogenous LP is usually good after the discontinuation of exposure. Complications such as pulmonary fibrosis, infection, or excavation can occur especially in case of persistent chronic exposure but have also been described in acute forms despite treatment [6].

## Abbreviations

LLAM: Lipid-laden alveolar macrophage
LP: Lipoid pneumonia.
